# Parental genetic material and oxygen concentration affect hatch dynamics of mouse embryo in vitro

**DOI:** 10.1186/s12958-018-0356-8

**Published:** 2018-04-21

**Authors:** Shaoquan Zhan, Shanbo Cao, Hongzi Du, Yuan Sun, Li Li, Chenhui Ding, Haiyan Zheng, Junjiu Huang

**Affiliations:** 1 Center for Reproductive Medicine, Key Laboratory for Reproductive Medicine of Guangdong Province, Key Laboratory for Major Obstetric Diseases of Guangdong Province, and Key Laboratory for Reproduction and Genetics of Guangdong Higher Education Institutes, the Third Affiliated Hospital of Guangzhou Medical University, Guangzhou, 510150 China; 2 Beijing Acorndx Biotechnology Co. Ltd, Beijing, 100176 China; 3 Key Laboratory of Reproductive Medicine of Guangdong Province, School of Life Sciences and the First Affiliated Hospital, Sun Yat-sen University, Guangzhou, 510275 China; 4 Guangzhou, People’s Republic of China

**Keywords:** Hatch, Fertilized embryo, Parthenogenetic embryo, Oxygen, Zona pellucida

## Abstract

**Background:**

Hatching is crucial for mammalian embryo implantation, since difficulties during this process can lead to implantation failure, ectopic pregnancy and consequent infertility. Despite years of intensive researches, how internal and external factors affecting embryo hatch are still largely unclear.

**Methods:**

The effects of parental genetic material and oxygen concentration on hatch process were examined. Fertilized and parthenogenetic mouse preimplantation embryos were cultured in vitro under 5 and 20% oxygen for 120 h. Zona pellucida drilling by Peizo micromanipulation were performed to resemble the breach by sperm penetration.

**Results:**

Firstly, parthenogenetic embryos had similarly high blastocyst developmental efficiency as fertilized embryos, but significantly higher hatch ratio than fertilized embryos in both O_2_ concentrations. 5% O_2_ reduced the hatch rate of fertilized embryos from 58.2 to 23.8%, but increased that of parthenogenetic embryos from 81.2 to 90.8% significantly. Analogously, 5% O_2_ decreased the ratio of *Oct4*-positive cells in fertilized blastocysts, whereas increased that in parthenogenetic blastocysts. Additionally, 5% O_2_ increased the total embryonic cell number in both fertilized and parthegenetic embryos, when compared to 20% O_2_, and the total cell number of fertilized embryos was also higher than that of parthegenetic embryos, despite O_2_ concentration. Real-time PCR revealed that the expression of key genes involving in MAPK pathway and superoxide dismutase family might contribute to preimplantation development and consequent blastocyst hatch in vitro. Finally, we showed that fertilized and parthenogenetic embryos have diverse hatch dynamics in vitro, although the zona pellucida integrity is not the main reason for their mechanistic differences.

**Conclusion:**

Both parental genetic material and O_2_ concentration, as the representative of intrinsic and extrinsic factors respectively, have significant impacts on mouse preimplantation development and subsequent hatch dynamics, probably by regulating the gene expression involving in MAPK pathway and superoxide dismutase family to control embryonic cell proliferation and allocation of ICM cells.

**Electronic supplementary material:**

The online version of this article (10.1186/s12958-018-0356-8) contains supplementary material, which is available to authorized users.

## Background

Hatching is a process essential for mammalian embryo implantation, in which the embryo at blastocyst stage escapes from its zona pellucida (ZP), an outer shell which is composed of glycoproteins and is responsible for the preventation of polyspermy and ectopic pregnancy [1–3]. The mechanism regulating the preimplantation embryos when and where to hatch is very critical for implanting in uterus exactly and successfully, since difficulties during this process can lead to implantation failure, ectopic pregnancy and consequent infertility [4, 5].

Many factors have been proved to function in embryo implantation in the past few decades, including embryonic cell number, trypsin-like proteinases, progranulin, prostanoid pathways, uterine function and the leukaemia inhibitory factor, zygote genomic expression and epigenetic changes, such as DNA methylation and histone deacetylation [6–14]. However, none of them has been identified as the determinant factor for embryo hatching. Additionally, the external culture environment is regarded as a key factor for preimplantation embryo growth in vitro, one of which is the oxygen (O_2_) concentration [15]. O_2_ plays an important role in the regulation of embryonic gene expression, allocation of cells to inner cell mass (ICM) and trophectoderm (TE) lineages and subsequent fetal development [16, 17]. Recent studies have showed that low O_2_ (5%) can promote embroyonic cell proliferation and reduce apoptosis, whereas it inhibit trophoblast cell invasion [17–25]. Conversely, other studies argued that there are no beneficial effects of 5% O_2_ culture on embryo subsequent development, such as implantation rate, pregnancy rate and fetal weight, when compared with atmospheric O_2_ culture [19, 26, 27]. It is also still controversial whether low O_2_ concentrations can be adopted as the standard for human embryo culture, at least for blastocyst formation. Thus, one of aims in our current study is to find out the relationship between O_2_ concentration and embryo hatching ability in vitro.

Furthermore, many oxygen-regulated genes have been proved to function in embryonic development and metabolish [9, 28], whereas it remains poorly understood whether there are any oxygen-regulated imprinted genes, connecting with the process of blastocyst hatch. Parthenogenetic embryos produced in vitro enable us to study the effect of maternally expressed genes in preimplantation embryos, since it is still unknown how parental genetic background influence embryo hatching [29, 30].

Besides that, sperm’s penetration through the ZP during fertilization, triggers the exocytosis of cortical granules in oocytes and biochemically alters the structure of ZP, thus making it impermeable to additional sperm, to prevent polyspermy [31–34]. So the integrity of ZP structure in normally in vivo fertilized embryos, is distinctly different from that in parthenogenetic embryos activated by chemicals in vitro, which is intact and completely hermetical. This provides us a powerful tool to study the effects of physical structural changes of ZP on embryo hatching process.

Therefore, this study aims to determine the effects of oxygen concentrations, parental genetic material, and integrity of ZP structure on blastocyst hatch, and tries to find out the molecular mechanisms regulating the blastocyst hatch, by utilizing fertilizated and parthenogenetic mouse embryos collected and cultured in vitro.

## Methods

Unless otherwise specified, all reagents were obtained from Sigma Chemical Co. (St. Louis, MO).

### Animal care and ethics statement

B6C3F1 mice were maintained in an environmentally controlled room at 22 ± 1 °C, under a light cycle of 14 h light/10 h dark, with free access to food and water in animal facility of specific pathogen-free (SPF) in Sun Yat-sen University. All experimental protocols involving the handling of mice were approved by the Institutional Animal Care and Use Committee of Sun Yat-sen University (Approval No. IACUC-2014-0102), People’s Republic of China.

### Collection of mouse oocytes and zygotes

Collection and culture of zygotes were performed as previously described [35–37]. B6C3F1 female mice aged at 5–8 weeks (weighed 16–19 g) were superovulated with 5 IU pregnant mare serum gonadotropin by intraperitoneal injection (PMSG, Calbiochem, La Jolla, CA), followed by 5 IU human chorionic gonadotrophin (hCG) 46–48 h later. Female mice were mated individually with B6C3F1 males of proven fertility, and successfully mated females indicated by a vaginal plug in the next morning, were used for collecting zygotes (day 0.5) at 20–21 h after hCG injection. Oocytes enclosed in cumulus masses were collected from oviduct ampullae 14 h after hCG injection. Cumulus cells were removed by pipetting after brief incubation in 0.03% hyaluronidase prepared in potassium simplex optimized medium (KSOM) containing 14 mM Hepes and 4 mM sodium bicarbonate (HKSOM). After removal of cumulus cells, zygotes and oocytes were then washed and incubated in 50 μL droplets of pre-equilibrated KSOM supplemented with nonessential and essential amino acids and 2.5 mM hepes (KSOM_AA_) and then covered with mineral oil pending further treatments. Zygotes and Oocytes culture and all subsequent embryonic culture experiments were carried out at 37 °C in a humidified atmosphere of 6.0% CO_2_ in air. All manipulations in vitro were performed at 36–37 °C on heated stages or in incubators. Embryos normally cleaved to two-cells at day 1–1.5, and formed morulae at day 2.5 (48 h in culture). Early blastocysts were formed at day 3.5 (72 h in culture), and late expanded blastocysts at day 4.5 (96 h in culture). Hatched blastocysts appeared at day5.5 (120 h in culture).

### Parthenogenetic activation of oocytes

Freshly ovulated metaphase II oocytes were collected from the ampullae 14 h after hCG injection, and cumulus cells were removed as described above. Diploid parthenogenetic embryos were produced by activation of oocytes with SrCl_2_ and cytochalasin D in 20% O_2_, as described before [30].

### Embryo culture in vitro

Both zygotes and parthenogenetic embryos were cultured in groups of 20–30 embryos per 50 μL droplet of KSOM_AA_ overlaid with embryo-tested mineral oil. Embryos were cultured in humidified atmospheres (Thermo) with 5% or 20% O_2_. Culture plates (35-mm petri dish; Corning Inc., Corning, NY) were prepared and equilibrated in the incubator 1 h before embryos collection. All control groups were made in the same day and the same treatment groups were cultured in the same dish with distinct makers. In all experiments, embryos were cultured for 5 days in vitro, and observed respectively at 24, 72, 96 and 120 h in vitro and graded for the stage of development including blastula formation and hatch.

### Embryo hypotonicity

The embryos culured for 48 h were transferred into a 20 μL droplet of pure water under mineral oil in 35 mm Petri dish directly, and immediately observed under Zeiss inverted microscope for live image.

### Zona pellucida drilling by Peizo micromanipulation

Ovulated metaphase II oocytes were collected at 14 h after hCG, and transferred into HKSOM microwell. A tiny breach (about 1 μm) on ZP was drilled with a pronuclear injection pipette or a 5 μm blunt-end pipette by Piezo micromanipulator.

### Live image with embryo

Each 4 un-hatched blastocysts cultured for nearly 96 or 120 h in vitro were transferred into 2 μL HKSOM medium covered with mineral oil in a 35 mm coated glass bottom microwell dish (MatTek corporation). The dish was put on a heating plate controlled by a temperature controller (Medical systems corp. Model: TC-202A) to keep 37 °C. Zeiss inverted microscope and AxionVision LE software were used to make live image.

### Immunofluorescence (IF) staining

Embryos were washed twice in phosphate buffered saline containing 0.1% polyvinyl pyrrolidone (PBS-PVP), then fixed in freshly prepared 3.7% paraformaldehyde in PBS-PVP (pH 7.4) for 15 min at 4 °C, permeablized in 0.1% Triton X-100 in blocking solution (3% goat serum in PBS-PVP) for 30 min, washed three times, and then left in blocking solution for 1 h. Embryos were incubated at 4 °C overnight with anti-*Oct*4 mouse monoclonal antibody (sc5279, santa cruz) diluted 1:50 in blocking solution, washed, and then incubated for 1 h with secondary antibodies conjugated to Alexa Fluor 568 (Molecular Probes) diluted 1:100 in blocking solution. Embryos were washed, and mounted onto a slide under a coverslip in the Vectashield with 0.2 μg/mL Hoechst 33342 mounting medium. Alexa Fluor-labeled *Oct*4 and Hoechst-labeled nuclei were observed with a Leica inverted fluorescence microscope.

### Detection of apoptosis by TUNEL assay

Embryos were collected, washed three times in PBS-PVP, and fixed in 3.7% paraformaldehyde overnight at 4 °C. Nuclear DNA fragmentation was detected by the TUNEL method using the In Situ Cell Death Detection Kit (Fluorescein, Roche Applied Science) according to the manufacturer’s instructions, and nuclei were counterstained with propidium iodide (PI, 50 μg/mL, Sigma). Fluorescence was detected using a Leica inverted fluorescence microscope.

### Real-time PCR relative quantitation of gene expression

Thirty blastocysts from both fertilized and parthenogenetic groups cultured in 5% or 20% O_2_ for 96 h in vitro were collected and used for mRNA extraction with RNeasy micro Kit (Qiagen, 74004). Total mRNA was subjected to cDNA synthesis using Reverse Transcription System (Toyobo). Primers for amplification of all the selected genes were designed using GeneTool Lite 1.0 (BioTools) (see Additional file 1). Polymerase chain reaction (PCR) was performed with ABI Prism 7900 Sequence Detection System (Applied Biosystems) using SYBR Green Real-time PCR Master Mix (Toyobo, QPK-201). Gene expression levels were analyzed and normalized to housekeeping gene β-actin as the reference gene.

### Statistical analysis

Percentages were transformed using arcsin transformation. Unless otherwise indicated, percentage transformed data, cell number, embryo hatch time and real-time PCR data were analyzed by one-way analysis of variance (ANOVA) and means were compared by Fisher’s protected least-significant difference (PLSD) using the StatView software from SAS Institute Inc. (Cary, NC). Significant difference was defined as *P* < 0.05.

## Results

### Both the oxygen concentration and genetic material affect the blastocyst hatch process in vitro

To assess the effects of oxygen concentration and genetic material on embryo development and hatch process, we carried out four combinatorial treatments in a two-factor design by using the in vitro model of embryo culture, in which both fertilized zygotes from naturally mated female mice and parthenogenetic embryos from chemically activated MII oocytes were cultured for 120 h in 5 and 20% O_2_ respectively.

**Fig. 1:**
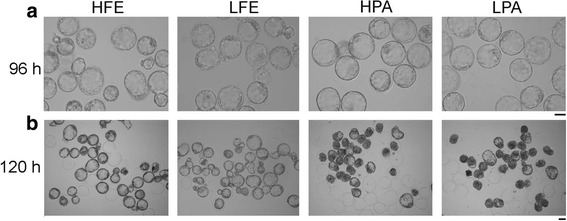
Morphology and hatch process of embryos cultured in vitro. **a** Morphology of fertilized and parthenogenetic embryos cultured in vitro for 96 h in 20% or 5% O_2_. Bar = 50 μm. **b** Morphology of fertilized and parthenogenetic embryos cultured in vitro for 120 h in 20% or 5% O_2_. Bar = 50 μm. HFE and LFE, fertilized embryos cultured in 20% and 5% O_2_ respectively; HPA and LPA, parthenogenetic embryos cultured in 20% and 5% O_2_ respectively

No detectable differences were observed in the blastocyst rates (around 97%) and morphology, among these four groups cultured for 96 h (Table 1 and Fig. 1a). However, the hatch rates among these groups showed significant differences after culture for 120 h (Table 1 and Fig. 1b). About 58.2% of fertilized embryos in 20% O_2_ hatched, which was notably higher than those in 5% O_2_ (23.8%, Table 1 and Fig. 1b). Conversely, the percentage of hatched parthenogenetic blastocysts in 5% O_2_ (90.8%) was significantly increased, compared with that in 20% O_2_ (81.2%, Table 1 and Fig. 1b). These results demonstrate that the oxygen concentration does not affect the development of either fertilized or parthenogenetic embryos to blastocyst (day 3), but influences the blastocyst hatch following blastulation (days 5). Interestingly, 5% O_2_ reduced the hatch ratio of fertilized embryos, but increased that in parthenogentic embryos, suggesting that there are distinct mechanistic differences between fertilized and parthenogentic embryos in regulating the effects of oxygen on blastocyst hatch. Moreover, the hatch ratio of parthenogenetic embryos was markedly higher than that of fertilized embryos, regardless of oxygen concentration (Table 1), implying a significant influence of parental genetic background on blastocyst hatch.

**Table 1:** Development potential of fertilized and parthenogenetic embryos cultured in 5 and 20% oxygen in vitro

Groups	Oxygen concentration %	Replicates No.	Total embryos No.	96 h	120 h
				No. (%) Blastocysts^+^	No. (%) Hatched Blastocysts
FE	20	7	324	312 (96.2 ± 1.4)	179 (58.2 ± 4.2)^a^
	5	7	350	337 (97.3 ± 1.1)	83 (23.8 ± 2.6)^b^
PA	20	9	346	336 (97.6 ± 0.8)	278 (81.2 ± 2.7)^c^
	5	9	346	335 (96.8 ± 1.0)	306 (90.8 ± 1.8)^d^

*FE* fertilized embryos, *PA* parthenogenetically activated embryos

^+^Mean ± SEM

^a^ vs. ^b^, *p* < 0.001; ^a^ vs. ^c^, *p* < 0.01;^c^ vs. ^d^, *p* < 0.05; ^b^ vs. ^d^, *p* < 0.001

### The allocation of ICM cells is affected diversely in fertilized and parthenogenetic blastocysts in vitro

**Fig. 2:**
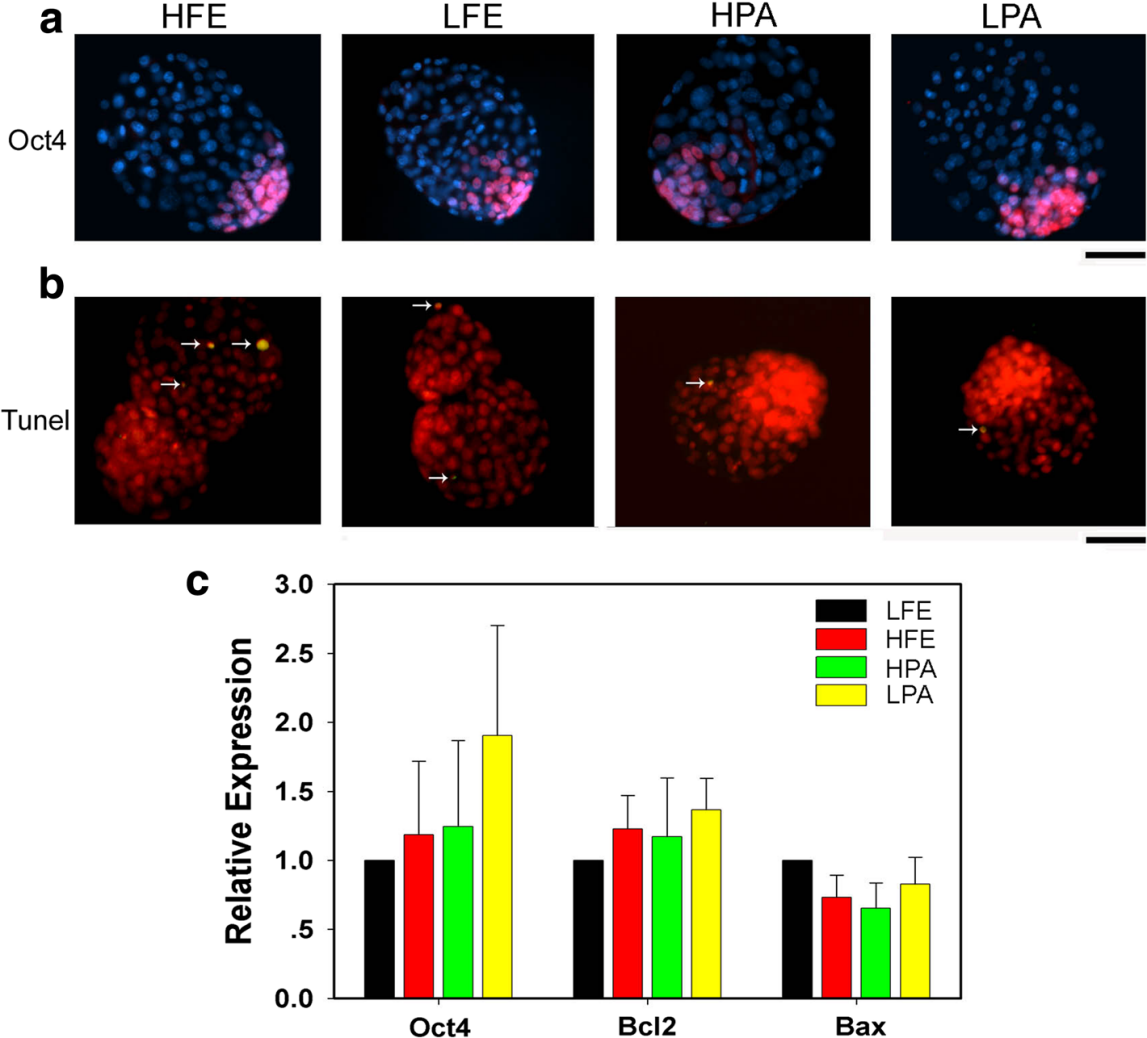
Embryonic cell differentiation and apoptosis in blastocysts cultured in vitro. **a** Immunofluorescent images showing *Oct*4*-*positive cells in fertilized and parthenogenetic blastocysts cultured for 120 h in 5% or 20%, indicative of inner cell mass (pink), with nuclei stained with Hoechst (blue). Bar = 50 μm. **b** Representative merged images showing apoptotic cells (yellow, white arrows) in fertilized and parthenogenetic blastocysts cultured for 120 h in 5% or 20% by TUNEL assay, with nuclei stained with PI (red). Bar = 50 μm. **c** Quantification of *Oct*4 and apoptotic genes, including *Bcl2* and *Bax*, in fertilized and parthenogenetic embryos cultured for 96 h. Relative mRNA expression levels are determined by real-time PCR and normalized to β-actin as internal reference, with the average value of LFE groups as control set to one and compared across groups. Data are presented as mean ± SEM. LFE and HFE, fertilized embryos cultured in 5% and 20% O_2_ respectively; LPA and HPA, parthenogenetic embryos cultured in 5% and 20% O_2_ respectively

To better understand the mechanism regulating embryo hatch, immunostaining for *Oct*4 protein, a crucial marker for ICM and embryonic stem cells (ESCs), and TUNEL assay were performed to check the cell allocation, apoptosis, and total embryonic cell number in these blastocysts among groups after culture for 120 h. There were no overt differences for the number of *Oct*4-positive cells in either fertilized or parthegenetic groups, when embryos cultured in 20% O_2_ was compared with those in 5% (Table 2 and Fig. 2a). Likewise, we found no any change for the number of apoptotic cells among groups, with less than two apoptotic cells observed in all groups of blastocysts by TUNEL assay (Table 2 and Fig. 2b). Congruently, when they were cultured for 96 h, that is, prior to hatching, embryos among groups had a comparable mRNA expression level of both *Oct*4 and apoptosis-associated genes, *Bcl2* and *Bax,* confirmed by real-time PCR analysis (Fig. 2c).

**Table 2:** Cell allocation and apoptosis in fertilized and parthenogenetic embryo cultured in vitro for 120 h

Groups	No. embryos	120 h Blastocysts^+^			No. embryos	120 h Blastocysts
		No. total cells	No. *Oct4* positive cells	*Oct4* positive cells/Total cells (%)		No. apoptosis cells
HFE	37	116.0 ± 3.2^a^	25.2 ± 1.2	21.7 ± 0.8^e^	14	1.9 ± 0.6
LFE	39	129.4 ± 4.4^b^	24.5 ± 1.2	18.8 ± 0.6^f^	13	1.2 ± 0.6
HPA	49	105.7 ± 2.6^c^	23.2 ± 1.0	21.9 ± 0.7^e^	11	1.4 ± 0.4
LPA	60	112.6 ± 2.7^acd^	27.6 ± 0.9	24.7 ± 0.6^g^	12	1.3 ± 0.4

*HFE and LFE* fertilized embryos cultured in 20 and 5% oxygen, *HPA and LPA* parthenogentic embryos cultured in 20 and 5% oxygen

^+^Mean ± SEM

^a^ vs. ^b^, *p* < 0.01; ^a^ vs. ^c^, *p* < 0.05; ^b^ vs. ^c^, *p* < 0.0001; ^b^ vs. ^d^, *p* < 0.001; ^c^ vs. ^d^, *p* < 0.05; ^e^ vs. ^f^, ^g^ *P* < 0.01; ^f^ vs. ^g^, *P* < 0.01

However, the ratio of *Oct*4-positive cells in each embryo after culture for 120 h varied considerably among these groups, due to a marked difference in the total embryonic cell number (Table 2). When compared with 20% O_2_, obviously, 5% O_2_ decreased the ratio of *Oct*4-positive cells in fertilized blastocysts, whereas increased that in pharthenogenetic blastocysts. This result resembles the data on hatch ratio (Table 1), and thus suggests a possible regulatory role for the allocation of ICM cells in blastocyst hatch. Furthermore, we found that the total cell number of fertilized embryos was higher than that of parthegenetic embryos in both 5% and 20% O_2_ (Table 2), and that 5% O_2_ increased the total cell number in both fertilized and parthenogenetic embryos, comparing to 20% O_2_ (Table 2). These data unambiguously illustrates the importance of both the parental genetic material and oxygen concentration on preimplantation development, and thus subsequent embryo hatch.

### Oxygen concentration affects the gene expression in fertilized and parthenogenetic blastocysts before hatching

**Fig. 3:**
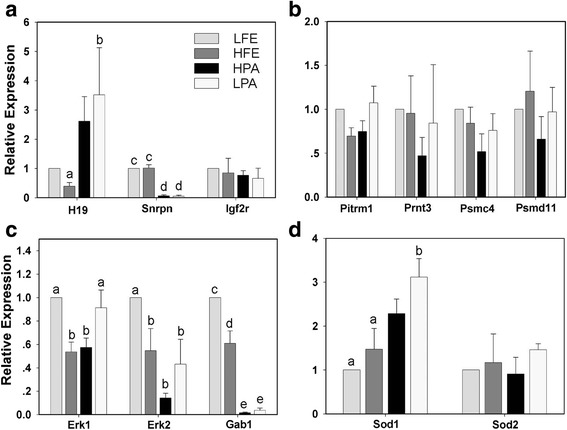
Quantification of key genes expression in embryos cultured in vitro for 96 h. **a** Imprinted genes expression. **b** Proteinase genes expression. **c** MAPK signal pathway genes expression. **d** Superoxide dismutase genes expression. LFE and HFE, fertilized embryos cultured in 5% and 20% O_2_ respectively; LPA and HPA, parthenogenetic embryos cultured in 5% and 20% O_2_ respectively. Gene-expression data are determined by real-time PCR and normalized to the levels of β-actin mRNA, with the average value of LFE groups as controls set to one and compared across groups. Data are presented as mean ± SEM, with letters indicating differences between groups, a vs. b, *p* < 0.05; c vs. d, *p* < 0.001; c vs. e, *p* < 0.0001; d vs. e, *p* < 0.0001

To find the key genes involved in the control of embryo hatch, several important genes involving in different cellular events were analyzed by real-time PCR, using fertilized and parthenogenetic blastocysts cultured in vitro for 96 h under 5% or 20% O_2_. Firstly, the expression patterns of three common imprinted genes in these four groups differed greatly as following: the expression of maternally-expressed *H19* was significantly increased but paternally-expressed Snrpn notablely reduced in both parthenogenetic embryo groups, when compared with fertilized embryo groups, and the expression of *Igf2r* was not changed among groups (Fig. 3a), indicating that their expressions are influenced only by the parental genetic background, rather than oxygen concentration. Secondly, the proteinase genes, *Pitrm1*, *Prnt3*, *Psmc4* and *Psmd11*, which were reported to function in regulating the hatch process [7, 38], demonstrated a similar expression level in all groups (Fig. 3b). Given that the mitogen-activated protein kinase (MAPK) signal pathway plays a key role in embryonic cell division [39], then, the expressions of three important regulating genes in this pathway were confirmed including *Erk1*, *Erk2* and *Gab1*, since we have found a significant change in the total cell number among groups (Table 2). As expected, the expression of all three genes in fertilized embryo groups were significantly increased in 5% O_2_ group (Fig. 3c). Similarly, in parthenogenetic embryo groups, *Erk1* expression significantly rised and *Erk2* and *Gab1* slightly increased in 5% O_2_ group (Fig. 3c). What’s more, *Gab1* expression was significantly reduced in parthenogenetic embryos in both 5 and 20% O_2_, when compared to paralleled fertilized embryos (Fig. 3c), showing a expression pattern similar to imprinted gene *Snrpn*. Finally, we checked the expression of *Sod1* and *Sod2*, two menbers from superoxide dismutases (SODs) family, which is the first and most important line of antioxidant enzyme defense systems against reactive oxygen species (ROS) and can thus help embryo developing well in vitro [40, 41]. While there was no difference found among all groups for *Sod2* expression, slightly higher expression level of *Sod1* was observed in parhtenogenetic embryos, when compared with fertilitzed embryos (Fig. 3d). There results clearly illuminate that oxygen concentration affects the gene expression in fertilized and parthenogenetic embryos, suggesting a potential role of MAPK in regulation of the preimplantation development and consequent blastocyst hatch in vitro*.*

### Sperm-drilling breach is not the main reason for different hatch dynamics between fertilized and parthenogenetic embryos

**Fig. 4:**
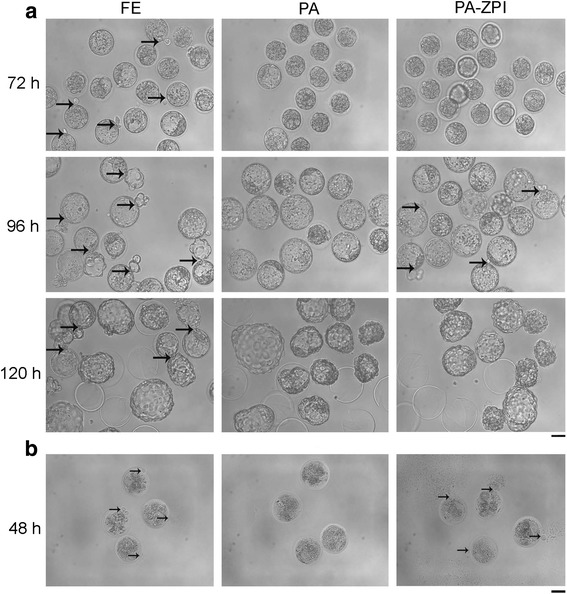
Different hatch patterns between fertilized and parthenogenetic embryos cultured in vitro. **a** Morphology of fertilized, normal and ZP-drilled parthenogenetic embryos cultured in vitro for 72, 96 and 120 h. Thick and black arrows indicated the place where embryos began to hatch. **b** Hypotonicity treatments with embryos cultured for 48 h. Thin and black arrows indicated the place where cytoplasm permeated out. FE, fertilized embryos cultured in 20% O_2_; PA, normal parthenogenetic embryos cultured in 20% O_2_; PA-ZPI, parthenogenetic embryos with a breach about 5 μm on ZP, drilled by a way like pronuclear injection with Piezo micromanipulator and cultured in 20% O_2_. Bar = 50 μm


To furthur elucidate how the embryo hatch was affected in both fertilized and parthenogenetic embryos, we monitored the embryo hatch process by living imaging and found that fertilized embryos spend about 42 h completing the hatch process, while parthenogenetic embryos spend only 6 h (see Additional files 2 and 3, Table 3). In detail, we found that fertilized embryos started hatching at 72 h and about 43% completed hatching at 120 h in 20% O_2_ (Fig. 4a and Table 3). Nevertheless, none of the 71% parthonegenetic embryos started to hatch until 120 h, without any signs observed at 72 or even at 96 h (Fig. 4a and Table 3), indicating a significant difference in the hatch dynamics between fertilized blastocysts and parthonegenetic blastocysts.

**Table 3:** Hatched percentage and time of different types of embryo

Embryo types	Diameter of hole on ZP	No. embryos	96 h Blastocysts	120 h Hatched Blastocysts (%)	Total Hatched time (repeats)
FE	Sperm-size	23	23	10 (43)^a^	41.7 ± 5.1^c^ (9)
PA	None	24	24	17 (71)^b^	6.0 ± 1.8^d^ (10)
PA-ZPI	~ 1 μm	10	10	8 (80)^b^	
PA-ZPI	~ 5 μm	20	20	15 (75)^b^	

^a^ vs. ^b^, *p* < 0.05; ^c^ vs. ^d^, *p* < 0.0001

**Additional file 2:** Hatch process of fertilized embryos in vitro. Fertilized embryos started hatching in 72 h and then spent about 42 h emerging from it gradually and slowly until completely hatched in 120 h. (MP4 1099 kb)



**Additional file 3:** Hatch process of parthenogenetic embryos in vitro. Parthenogenetic embryos kept expansion until broken out suddenly and quickly within approximately 6 h after culture for 96 h. (MP4 283 kb)


Since sperm penetration through the oocyte ZP makes the resultant fertilized embryos different from parthenogenetic embryos in the physical structure of ZP, we wondered whether this divergence could also lead to marked differences in hatch process. To answer this question, we performed the hypotonic experiment using embryos cultured for 48 h. Significantly, the cytoplasm permeated out through a breach on ZP from all fertilized embryos after transferred into pure water, while no cytoplasm leaked out in parthenogenetic embryos (Fig. 4b, Additional files 4 and 5), indicating that the breach generating from sperm’s penetration may be the reason leading to two different hatched mechanisms.


**Additional file 4:** Hypotonicity of 48 h fertilized embryo in vitro. Cytoplasm came out from sperm-breach quickly after transferred into pure water. (MP4 333 kb)



**Additional file 5:** Hypotonicity of parthenogenetic embryo in vitro. No cytoplasm came out from parthenogenetic embryos after transferred into pure water. (MP4 341 kb)


In order to verify this possiblibity, a tiny breach (about 1 μm) was artificially made on the mature oocyte ZP using pronuclear injection method. After the oocytes were parthenogenetically activated and then cultured for 48 h, we found that no cytoplasm permeated out from the ZP-drilled parthenogenetic embryos in hypotonic experiment, and no hatching embryo was observed before 96 h, and 80% embryos (8/10, Table 3) hatched in 120 h, indicating the same hatch pattern as normal parthenogenetic embryos. One of the potential reasons for the above results is that the breach drilled by pronuclear injection pipette was much smaller than that generated by sperm penetration in fertilized embryo, which would block the cytoplasm outflowing in hypotonic experiment. Thus, a wider injection pipette (approximate 5 μm in blunt-end) was used to make a larger breach on oocyte ZP at similar size as sperm penetration in fertilized embryos. After hypotonic treatment at 48 h, the cytoplasm permeated out from ZP-drilled parthenogenetic embryos (Fig. 4b, Additional file 6). Furthermore, ZP-drilled parthenogenetic embryos started to hatch at 96 h, and 75% embryos (15/20) completed hatching in 120 h, which were similar to normal parthenogenetic embryos but significantly higher than fertilized embryos (Fig. 4a and Table 3). However, we found that the blastocysts did not come out from the aritificial breach on ZP, but kept expanding and then hatched out from another place of ZP suddenly and quickly, although some trophoderm cells had already came out through the sperm-like breach at 96 h (see Additional file 7). These results indicate that the sperm-like breach on ZP was not the main reason for the mechanistic difference of hatch between parthenogenetic and fertilized embryos.


**Additional file 6:** Hypotonicity of parthenogenetic embryo with 5 μm breach drilled on zona pellucid in vitro. Cytoplasm came out from artificial breach quickly as same as fertilized embryos after transferred into pure water. (MP4 330 kb)



**Additional file 7:** Hatch process of parthenogenetic embryo with 5 μm breach drilled on zona pellucid in vitro. Parthenogenetic embryos started hatching slightly from artificial breach in 96 h, but embryonic cells did not keep emerge from it. They kept expansion and broke out from another place on zona pellucid in a short time as normal parthenogenetic embryos did. (MP4 580 kb)


## Discussion

Uterus interacts with embryos and secretes lots of lytic factors to help embryo hatch and implant [42, 43]. Whereas, uterus is not the determinant issue for embryo hatch, since the embyos are able to hatch by themselves in vitro, through a way different from that developed in vivo [13, 44]. Assisted hatching (AH), an invasive technique aiming to facilitate embryo implantation by artificially manipulating zona pellucida in vitro, has been widely used, although its clinical efficacy remains controversial [4, 45–47]. Understanding molecular mechanisms involving in embryo hatching will enable the development of new interventions for embryo implantation and thus improve the human reproductive outcomes. Here, our results indicate that the mechanism of mouse embryo hatch is regulated by both intrinsic and extrinsic factors in vitro.

### Both oxygen concentration and parental genetic material affect preimplantation development and subsequent blastocyst hatching

Oxygen concentration is one of the most important extrinsic factors for embryo culture in vitro [15–17]. In our present study, we found that both fertilized and parthenogenetic embryos developed to blastocyst stage well in 5% and 20% O_2_ at 96 h, which means that oxygen concentration does not affect embryo developmental morphology, as reported in bovine [17], mouse [19], and human [26, 27]. However, after culture for 120 h, that is, after blastocyst formation, we found that 5% O_2_ increased the total cell number in both fertilized and parthenogenetic embryos, comparing to 20% O_2_. This result is accordant with a previous conclusion that 5% O_2_ is better for mouse embryo development [19, 22, 48, 49]. Moreover, in both 5% and 20% O_2_, the total cell number of fertilized embryos was significantly higher than that of parthegenetic embryos. These data indicate that both oxygen concentration and parental genetic background can regulate embryonic cell proliferation after blastulation, and thus affect the subsequent hatch process in vitro.

As for the effects of oxygen concentration on hatch, we found that 5% O_2_ reduced the hatch rate of fertilized embryos, which is consistent with Fischer’s report that bovine fertilized embryos have notablely higher hatched rate cultured under 20% O_2_ in KSOM medium [17]. On the contrary, we found that 5% O_2_ increased the hatch rate in parthenogentic embryos, which suggests that the mechanism regulating the effects of oxygen concentration on hatch process in parthenogentic embryos is distinctly different from that in fertilized embryos. Moreover, the hatch ratio of blastocysts showed significant differences among groups after culture for 120 h, and implied a significant effect of maternal genetic background on embryo hatch, revealed by the markedly higher hatch ratio observed in parthenogenetic embryos than fertilized embryos, regardless of oxygen concentration.

We suppose that 5% O_2_ increased the total embryonic cell number in both fertilized and parthenogenetic blastocysts, mainly by stimulating TE cells proliferation, since we did not find any difference in either embryo qualities, indicated by the similar number of apoptotic cells and camparable expression level of apoptosis related genes in all embryo groups, or embryonic cell differentiation, indicated by no changes for the expression level of pluripotent gene *Oct*4 and the number of *Oct*4*-*positive cells that are considered as ICM cells [50]. Nevertheless, comparing with 20% O_2_, the reason why 5% O_2_ inhibit fertilized embryos but promote pharthenogenetic embryos hatching remains unclear. In this study, we amazedly found that 5% O_2_ significantly decreased the ratio of *Oct*4-positive cells in fertilized blastocysts, whereas increased that in pharthenogenetic blastocysts. That is to say, both fertilized and parthenogenetic embryos with higher hatched rate, have higher ratio of *Oct*4-positive cells accordingly, thus indicating a potential close relationship between the allocation of ICM cells and subsequent blastocyst hatch. We speculate that embryos with higher ICM ratio likely secrete more factors, which may be benefiticial for embryo hatch [42, 43].

Taking into account that oxygen can regulate the expression of embryonic genes, and that low oxygen can affect the expression of oxygen-regulated genes in mouse and bovine embryos [9, 16, 28], we performed real-time PCR to analyze the expression levels of key genes that may be involved in the control of embryo hatch, by using fertilized and parthenogenetic blastocysts cultured in 5% and 20% O_2_ for 96 h, when the mRNA transcript expression level would be changed at this stage, if any of these genes is functioned in regulating embryo hatch. Since we found that the expression level of three common imprinted genes including *H19*, *Snrpn*, and *Igfr2*, are influenced only by the parental genetic background, as previously reported [16], further studies are required to better characterize whether there are any aother imprinted genes involving in the blastocyst hatch process. Also, our results showed no changes for all the four proteinase genes including *Pitrm1*, *Prtn3* and *Psmd11* as well as *Psmc*, indicating that these proteinase genes might not be impacted by oxygen concentration during embryo hatching, although they were demonstrated to be important for embrygenesis and embryo hatching in previous studies [7, 38].

However, we found that 5% O_2_ significantly indeed increased *Erk1* and *Erk2* expression in both fertilized and parthenogenetic embryos, which is one of the reasons why 5% O_2_ can promote embryonic cell proliferation, indicated by increased total embryonic cell number, when compared with 20% O_2_. Simultaneously, the expression level of *Gab1*, another gene in MAPK pathway, was significantly increased in fertilized embryos in both 5% and 20% O_2_, which accounts for their significantly higher total cell number, when compared to paralleled parthegenetic embryos. Furthermore, we analyzed the expression level of two genes from superoxide dismutases (SODs) family, and found no change for *Sod2* expression, whereas slight higher expression level of Sod1 was observed in parhtenogenetic embryos, when compared with fertilitzed embryos. Given that superoxide dismutases can protect against reactive oxygen species (ROS) [40, 41], we hence presume that reduced oxidative stress, due to increased sod1 expression, may be one of the mechanisms to faciliate embryo hatch, indicated by the higher hatch rate in parhtenogenetic embryos. But, this hypothesis might not be suitable for normal fertilized embryos, because of the distinct differences in mechanisms regulating the hatch process between fertilized and parthonegenetic blastocysts. Therefore, it will be meaningful to further explore whether antioxidants, such as N-Acety-L-Cysteine (NAC) or resveratrol [37, 51], can increase the hatch rate of human embryos and thereby improve the clinic outcomes, since the relationship between the oxidative stress and embryo hatch remains largely unknown.

### Sperm-like breach induces embryo hatch and genetic background determines the hatch mechanism

In our present study, markedly higher hatch rate observed in parthenogenetic embryos than fertilized embryos, regardless of oxygen concentration, resembled the result as previous reported [12]. Furthermore, when we use living imaging to monitor the hatch process in vitro, we amazedly found that fertilized embryos showed a hatch pattern different from parthenogenetic embryos. Fertilized embryos usually started hatching earlier and spent about 42 h to complete hatching, which was nearly seven times longer than parthenogenetic embryos that initiate hatching after 96 h. This result indicated that these two types of embryos had totally different mechanisms to regulate hatch, which might be caused by the genetic background and ZP structure.

On the one hand, Parental genetic background difference can affect TE cells function and make parthenogenetic embryo have much more rapid expansion ability [12], which thus lead to such a short time hatched. On the other hand, there is at least one breach on the ZP of fertilized embryos due to sperm penetration, making them different that of parthenogenetic embryos. The hypotonic experiment result showed that the fertilized embryos prefer to start hatching from sperm-driling gaps at about 72 h by plumping TE cells, thinning ZP and then breaking ZP, after which they continue to widen the ZP gap with “zona breaker” cells and the TE cells emerg to form dumb-bell shap like human embryos, and finally the whole embryo come out gradually [52, 53]. Unlike fertilized embryos, parthenogenetic embryos have hermetical and intact ZP, and they usually keep plumping TE cells and thinning ZP till 96 h or later, and then suddenly break ZP and come out quickly.

To identify whether it is caused by the change of ZP integrity, we tried to make a breach on parthegenetic embryo’s ZP artificially by Peizo micromanipulation [54]. No change happened when a tiny breach was made, whereas ZP-drilled parthenogenetic embryos started hatching at 96 h with a small mass of zona breaker cells when the breach size reached to 5 μm which is as large as sperm penetration. Unexpectedly, instead of keeping emerging as fertilized embryos, these cells stoped and the whole embryo hatched suddently and quickly from another place of ZP after long-term expansion, just like normal parthenogenetic embryos. These results indicate that the divergence in ZP structure is not the main reason for the different hatching patterns between fertilized and parthenogenetic embryos. These data suggest that the sperm-drilling breach just induces embryo hatching but embryo itself knows how to continue this process by an unknown mechanism that requires further investigation.

## Conclusion

In conclusion, the mechanism of mouse embryo hatch is regulated by both intrinsic and extrinsic factors in vitro. This result has significant clinical implications for the patients who requires AH during IVF treatment, meaning that, in addition to patients’ clinical indications, the embryo culture condition should be taken into consideration, when this technique is used to facilitate embryo implantation. O_2_ concentration had different influences on the expression of key genes involving in MAPK pathway and superoxide dismutase family between fertilized and pharthenogenetic blastocysts. The regulation of gene expression might control embryonic cell proliferation and the allocation of ICM cells, indicated by the total embryonic cell number and ratio of *Oct4*-positive cells, and thus leads to diverse blastocyst hatch dynamics in different parental genetic background. Therefore, human embryos awaiting AH should be cultured under the atmospheric O_2_ (20%) after fertilization, aiming to promote hatching and improve the reproductive outcomes, if the result of mouse study can be applied to humans. However, more animal and human clinical studies are required to clarify the mechanism underlying embryo hatching.

## Additional files

Additional file 1: Real time PCR primers for genes expression analysis of 96 h blastocysts. (DOC 41 kb)

## Abbreviations

AH: Assisted hatching
ANOVA: One-way analysis of variance
ESCs: Embryonic stem cells
hCG: Human chorionic gonadotrophin
HKSOM: KSOM containing 14 mM Hepes and 4 mM sodium bicarbonate
ICM: Inner cell mass
IF: Immunofluorescence
KSOM: Potassium simplex optimized medium
KSOM_AA_: KSOM supplemented with nonessential and essential amino acids and 2.5 mM hepes
MAPK: Mitogen-activated protein kinase
NAC: N-Acety-L-Cysteine
O_2_: Oxygen
PBS-PVP: Phosphate buffered saline containing 0.1% polyvinyl pyrrolidone
PCR: Polymerase chain reaction
PLSD: Fisher’s protected least-significant difference
ROS: Reactive oxygen species
SODs: Superoxide dismutases
SPF: Specific pathogen-free
TE: Trophectoderm
ZP: Zona pellucida
