# Supplementary material for: Parental genetic material and oxygen concentration affect hatch dynamics of mouse embryo in vitro
Source: Reprod Biol Endocrinol. 2018 Apr 21;16:39. doi: 10.1186/s12958-018-0356-8 (PMC5911372; doi:10.1186/s12958-018-0356-8)
Supplement: Supplementary file 7 — Real time PCR primers for genes expression analysis of 96 h blastocysts. (DOC 41 kb) [file 12958_2018_356_MOESM1_ESM.doc]

**Real time PCR primers for genes expression analysis of 96 hours blastocysts.**

| **Gene** | **Accession number** | **Primers sequence (**5'-3') | **Product size (bp)** |
| --- | --- | --- | --- |
| Oct4 | NM_013633 | F: GTTGGAGAAGGTGGAACCAA  R: CCAAGGTGATCCTCTTCTGC | 107 |
| Bax | NM_007527 | F: GCGGGCCCACCAGCTCTGAAC  R: GGGCGGCTGCTCCAAGGTCA | 102 |
| Bcl2 | NM_009741 | F：TCGCTACCGTCGTGACTTCGCAGAG  R：GGCCACAATCCTCCCCCAGTTCA | 108 |
| H19 | AK145379 | F: CGATTGCACTGGTTTGGA  R: TCAGACGGAGATGGACGA | 119 |
| Snrpn | NM_013670 | F: AGGCCCTACCCAGCAGGTCAT  R: GCGGGTACTGGCTTGGGGCTC | 144 |
| Igf2r | NM_010515 | F: AGGCCGGTCGGGATGGAGAG  R: GGGGGCTCCTGGGTAAATGTCAT | 104 |
| Pitrm1 | BC006917 | F: CCGGCGGACAAGCAGCAGATC  R: GGGAGGCAGGAGGCGTCTTGATGTT | 84 |
| Prtn3 | NM_011178 | F: GCCCCGTGTGCTGCAGGAACT  R: CGCCCGAGTCTCCGAAGCATATG | 116 |
| Psmc4 | [BC012708](http://www.ncbi.nlm.nih.gov/entrez/viewer.fcgi?db=nucleotide&val=15215224) | F: AGCCCTCAGAGCAAGCAAGACACT  R: CCGGGGCAGTTTTCTCAGGA | 136 |
| Psmd11 | NM_178616 | F: CAGGGAGGCAGACAGAAGCATTGA  R: CCCGGAGCTCTGCCCTGTAGTCT | 105 |
| Erk1 | NM_011952 | F: CCCCACCCCATTTTCCC TGACAG  R: CCCCACATCCAATCACCCACACA | 141 |
| Erk2 | NM_011949 | F: GCTGTCTGGTGGATGGGCTTTTTGT  R: TGCCGGTATCATCAAGCGCTCTG | 127 |
| Gab1 | NM_021356 | F: TCGACCGGATTTTCTACTTGG  R: GTCAGCGGCTTCACAGGAT | 118 |
| Sod1 | NM_011434 | F: TCGGCTTCTCGTCTTGCTCTCTCTG  R: CGCCCTTCAGCACGCACACC | 98 |
| Sod2 | NM_013671 | F: TCCTCCCAGACCTGCCTTACGACTA  R: CCGCGTGGTGCTTGCTGTGGT | 99 |
| β-actin | NM_007393 | F: TCCATCATGAAGTGTGACGT  R: GAGCAATGATCTTGATCTTCAT | 142 |
